# Supplementary material for: Live-cell imaging reveals the spatiotemporal organization of endogenous RNA polymerase II phosphorylation at a single gene
Source: Nat Commun. 2021 May 26;12:3158. doi: 10.1038/s41467-021-23417-0 (PMC8155019; doi:10.1038/s41467-021-23417-0)
Supplement: Supplementary file 1 — Supplementary information [file 41467_2021_23417_MOESM1_ESM.pdf]

Supplementary Information

Live-cell imaging reveals the spatiotemporal organization of endogenous RNA polymerase II phosphorylation at a single gene

Linda S. Forero-Quintero, William Raymond, Tetsuya Handa, Matthew N. Saxton, Tatsuya Morisaki, Hiroshi Kimura, Edouard Bertrand, Brian Munsky, and Timothy J. Stasevich

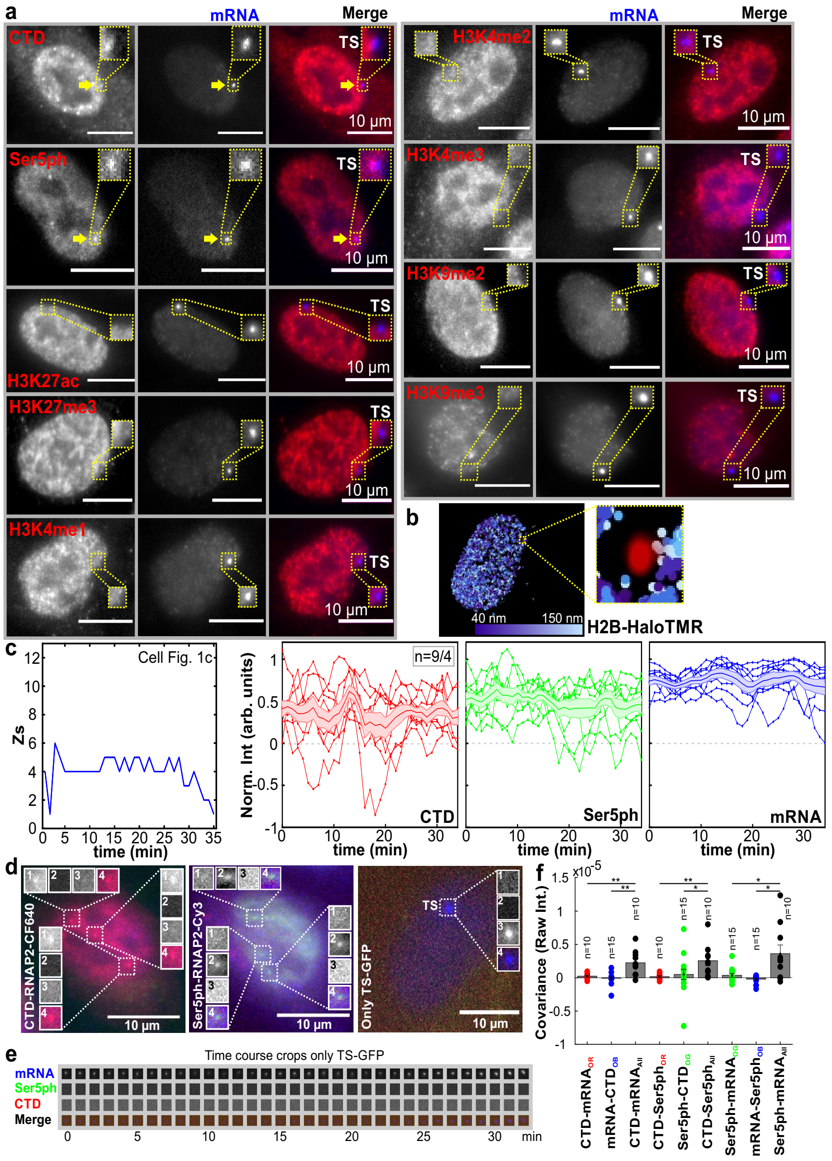

Sup. Fig. 1: **Immunostaining, single H2B tracking, and control experiments for photobleaching and bleed through. (Continues next page.)**

Sup. Fig. 1: **(a)** Immunostaining (red, left panels) of CTD-RNAP2 (n=11), Ser5ph-RNAP2 (n=10), histone H3K27ac (n=12), H3K27me3 (n=14), H3K4me1-3 (n=3) and H3K9me2-3 (n=3) at the HIV-1 transcription site (TS) marked by MCP-GFP (mRNA; blue, center panels), along with a merge (right panels). **(b)** Representative cell showing a mobility map of single H2B tracks. The blue scale shows the average frame-to-frame jump size (one frame every 43.86 ms) for each tracked molecule. The track corresponding to the transcription site is shown in red. The yellow dashed box displays a zoom-in around the transcription site region, where H2B is depleted (n=17 out of 28 cells in 3 total days). Control experiments for photo-bleaching showing **(c)** left panel, "best-Z" positions of the TS over time for the exemplary cell in Fig. 1c; right panels, normalized intensity over time for CTD-RNAP2 (red circles), Ser5ph-RNAP2 (green squares), and mRNA (blue diamonds) for all the cells recorded as in Fig. 1c,d. The shadow and the line in the middle represent the S.E.M and the average. **(d)** Images of cells from bleed-through control experiments. Left, a cell loaded with just Fab marking CTD-RNAP2 (CTD-RNAP2-CF640) displays endogenous puncta that are not the TS (designated Only Red "OR" spots; n=10); Middle, a cell loaded with just Fab marking Ser5ph-RNAP2 (Ser5ph-RNAP2-Cy3) displays endogenous puncta that are not the TS (designated Only-Green "OG" spots; n=15); Right, a cell without Fab in which the TS is marked solely by GFP-MCP binding mRNA (Only TS-GFP; Only Blue "OB"; n=15). Cropped images show the various "OR", "OG", and "OB" sites where the individual channels are separated and labeled as follows: (1) Red (CTD-RNAP2), (2) Green (Ser5ph-RNAP2), (3) Blue (mRNA), and (4) Merge. **(e)** Cropped images in a time course at an "OB" site demonstrates no bleed through of the mRNA channel into the other channels. **(f)** Covariance between all possible pairs of raw intensity signals is not significant at "OR", "OG", and "OB" sites, but is significant at the TS in cells containing all three signals (i.e. cells loaded with both Fab and expressing MCP-GFP; All). n=number of cells/independent experiments. Data are presented as mean values $\pm$ S.E.M. Significance was tested using a two-tailed Mann-Whitney U-test with $p \leq 0.0136$ (*) and $p \leq 0.0091$ (**).

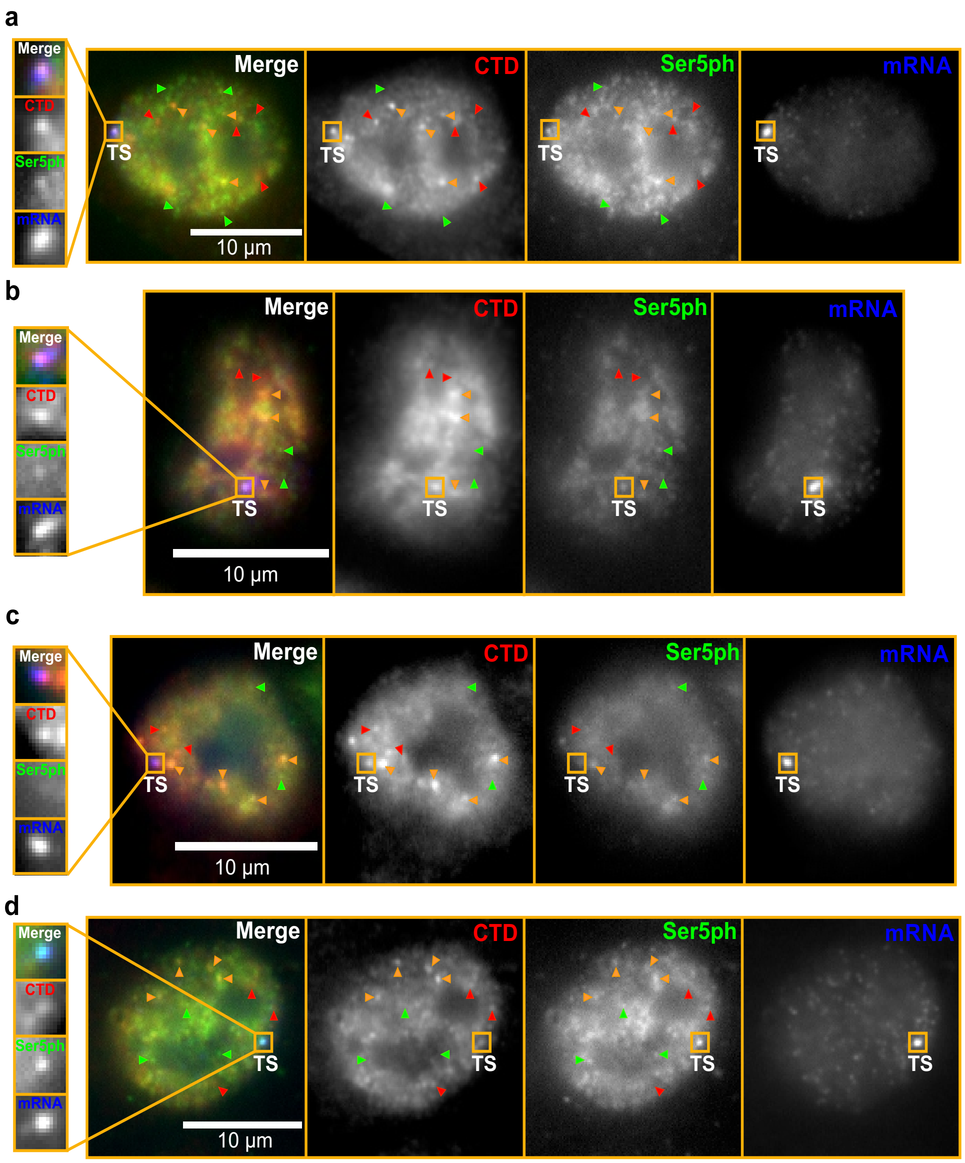

Sup. Fig. 2: **Fixed cells stained with our CTD- and Ser5ph-specific Fab.** Cells with distinct staining patterns. Some areas within cell nuclei are enriched with CTD-specific Fab (red arrows), other areas are enriched with Ser5ph-specific Fab (green arrows), while still other areas are enriched with both Fabs (orange arrows). At the HIV-1 transcription site (TS), we typically see both Fabs present **(a)**, n=20 out of 29 cells. However, on occasion we can find TSs in which Ser5ph-RNAP2 staining is relatively dim **(b & c)**, n=8 out of 29 cells, or, in very rare cases, CTD-RNAP2 staining is relatively dim **(d)**, n=1 out of 29 cells. This provides evidence that the signals are fluctuating.

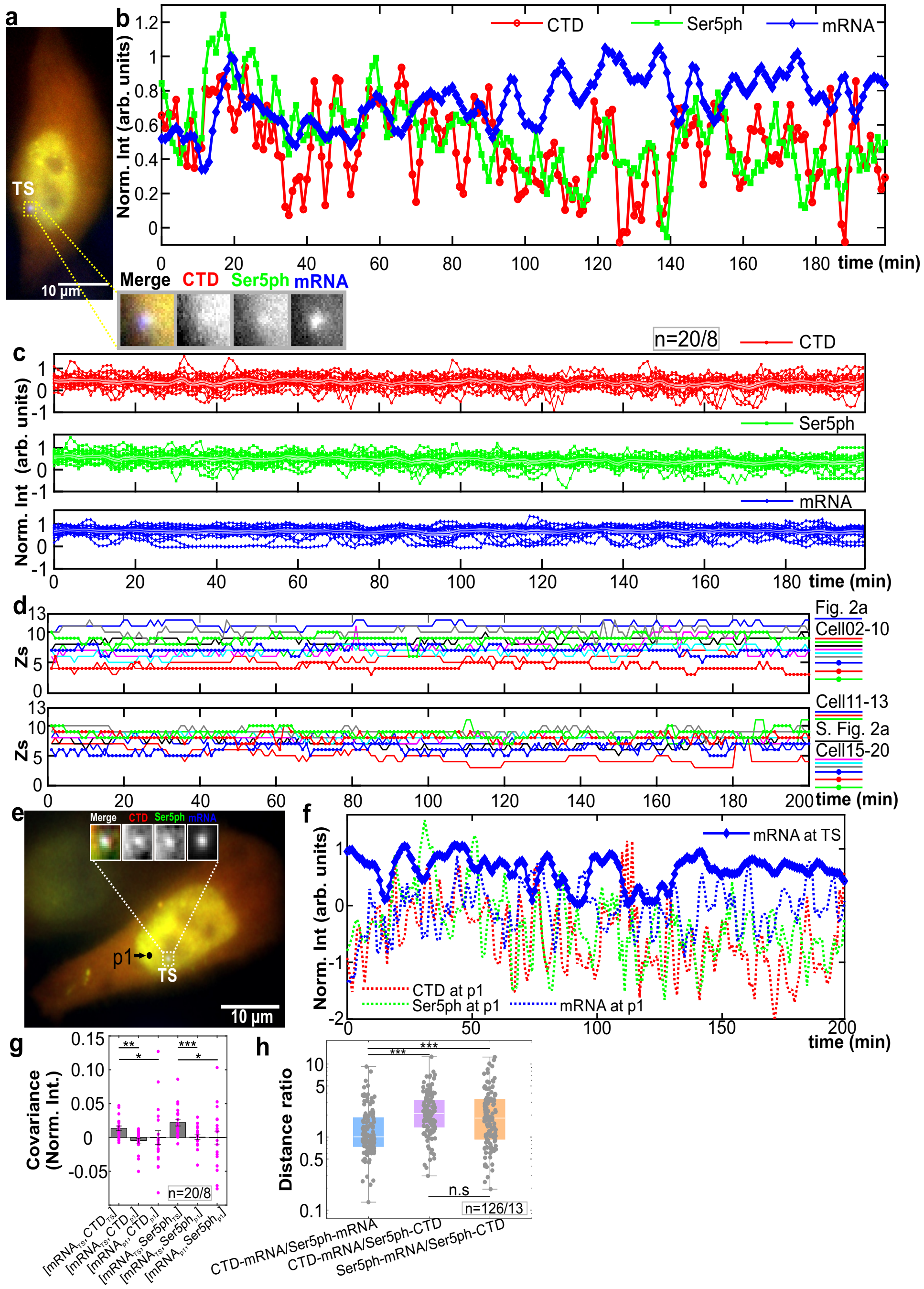

Sup. Fig. 3: **RNAP2 fluctuations at the HIV-1 reporter locus and off target. (Continues next page.)**

Sup. Fig. 3: **(a,b)** A cell with strong and persistent transcription has co-localized CTD-RNAP2 (red circles), Ser5ph-RNAP2 (green squares), and mRNA (blue diamonds) at the transcription site (TS), n=7 out of 20 cells. **(c)** Normalized signal intensities over time for all the cells analyzed as in b. The shadow and the line in the middle represent the S.E.M and the average, respectively. **(d)** Z-positions of all the cells quantified for transcription fluctuations. Each cell is represented with a different color/symbol (legend on the right). **(e)** Exemplary cell with periods of active and inactive transcription showing a control position (p1) near the transcription site, n=13 out of 20 cells. **(f)** Normalized intensity over time-target at an off-target position near the transcription site (p1; CTD-RNAP2, dashed red; Ser5ph-RNAP2 dashed green; mRNA, dashed blue) versus the mRNA signal at the transcription site (blue diamonds). **(g)** Covariance calculation between the normalized intensities of mRNA at the transcription site against CTD-RNAP2 or Ser5ph-RNAP2 at the transcription site and at p1. Data are presented as mean values $\pm$ S.E.M. n=number of cells/number of independent experiments. **(h)** Ratiometric distribution of the euclidean distances for CTD-RNAP2 and mRNA to Ser5ph-RNAP2 and mRNA (light blue), CTD-RNAP2 and mRNA to Ser5ph-RNAP2 and CTD-RNAP2 (light purple), and Ser5ph-RNAP2 and mRNA to Ser5ph-RNAP2 and CTD-RNAP2 (light orange) in all the cells analyzed. The line in the middle of each box represents the mean. The top and the bottom of the box represent the 75% and 25% quantiles, respectively. The middle region in the error bar at the bottom and the top represent the lower and upper whiskers, respectively. n=number of events/number of cells. Significance was tested using a two-tailed Mann-Whitney U-test with $p \leq 0.05$ (*), $p \leq 0.0015$ (**), and $p \leq 9.2091\times10^{-4}$ (***).

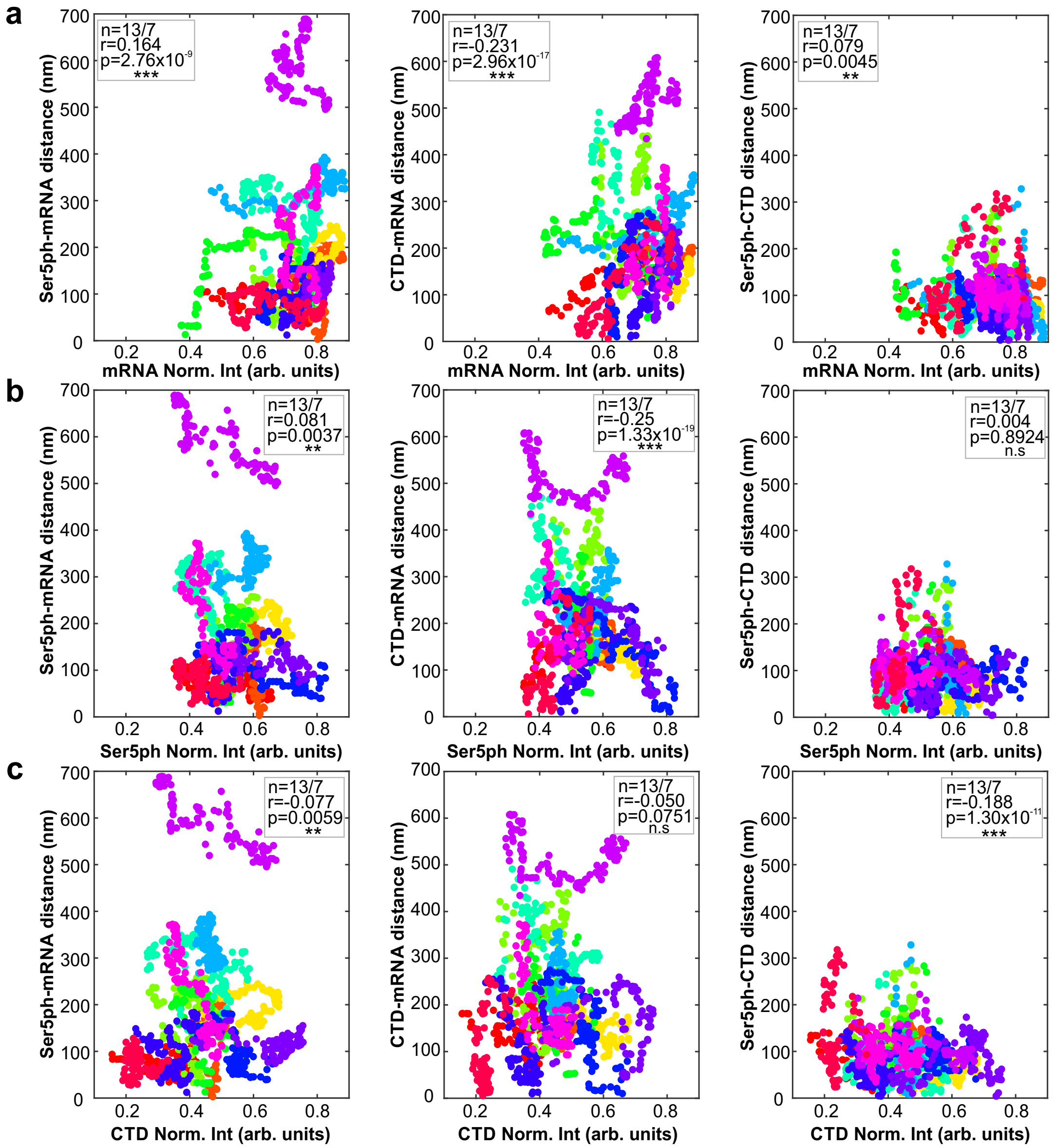

Sup. Fig. 4: **Euclidean distances distribution versus mRNA, Ser5ph-RNAP2, and CTD-RNAP2 normalized intensities.** Euclidean distance between Ser5ph-RNAP2 and mRNA (left panel), CTD-RNAP2 and mRNA (middle panel), and Ser5ph-RNAP2 and CTD-RNAP2 (right panel) versus the normalized intensities of **(a)** mRNA, **(b)** Ser5ph-RNAP2, and **(c)** CTD-RNAP2 for all the cells analyzed. Each cell corresponds to one color. n=number of cells/number of independent experiments. Correlation coefficient (r) and p-values (p) as, $p \leq 0.05$ (*), $p \leq 0.01$ (**), and $p \leq 0.001$ (***) were calculated using the "corrcoef" function in MATLAB.

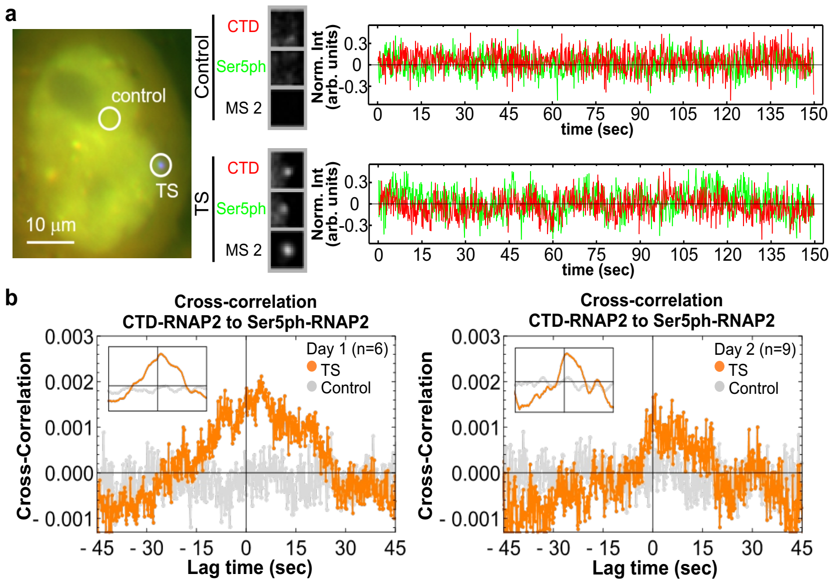

Sup. Fig. 5: **Fast-imaging experiments revealed a 3-6 sec time delay between CTD-RNAP2 and Ser5ph-RNAP2.** **(a)** (Left) Exemplary cell for fast imaging (150 msec/frame) for a total of 1000 time points (150 sec) in a single plane. Two positions are highlighted: the HIV-1 TS and a control nonspecific spot. (Center) Crops showing the CTD- (red), Ser5ph-RNAP2 (green), and MS2 mRNA (black) signals at the TS and control positions within the exemplary cell. (Right) Normalized intensity at the TS (bottom) and control (top) positions over time from the exemplary cell for CTD-RNAP2 (red), Ser5ph-RNAP2 (green). **(b)** Measured cross-correlation function $CC(\tau)$ between CTD-RNAP2 and Ser5ph-RNAP2 at the TS (orange circles) and control (gray circles) positions separated by experimental day. The inset shows a 50-frame rolling average to more easily identify the peak time delay between the two signals. In both cases, the cross-correlation peaks at a lag time of roughly 3-6 seconds.

Two State Bursting Model

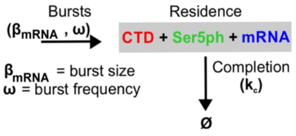

Parameter	Fit	
	Only mRNA	All
β	6.261	6.261
ω (min^{-1})	0.446	0.519
k_{ab} (min^{-1})	--------	--------
k_{esc} (min^{-1})	--------	--------
k_c (min^{-1})	0.180	0.209
# Parameters	3	3
Log likelihood	--------	25.242
BIC		53.192

Two State with unfixed k_{off}

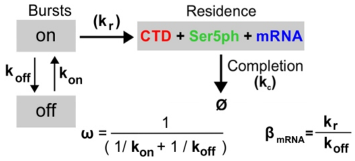

Parameter	Fit
β	6.2619
ω (min^{-1})	0.5183
k_r (min^{-1})	6112.9
k_{on} (min^{-1})	0.5168
k_{off} (min^{-1})	976.2
k_c (min^{-1})	0.2094
# Parameters	4
Log likelihood	25.241
BIC	54.094

Chosen Model

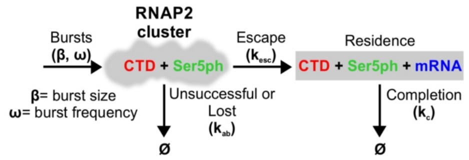

Parameter	Fit
β	15.405
ω (min^{-1})	0.434
k_{ab} (min^{-1})	0.778
k_{esc} (min^{-1})	0.666
k_c (min^{-1})	0.199
# Parameters	5
Log likelihood	14.558
BIC	33.632

Fractional Phosphorylation Model

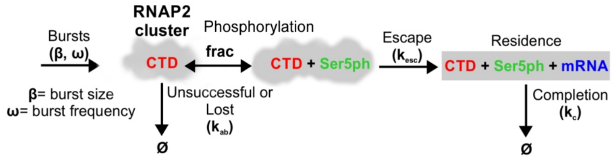

Parameter	Fit
β	15.407
ω (min^{-1})	0.434
k_{ab} (min^{-1})	0.783
k_{esc} (min^{-1})	0.667
k_c (min^{-1})	0.199
# Parameters	6
Fraction	1
Log likelihood	14.558
BIC	34.535

Phosphorylation Model

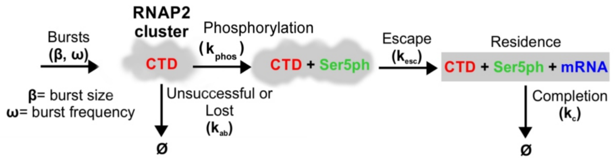

Parameter	Fit
β	15.427
ω (min^{-1})	0.434
k_{ab} (min^{-1})	0.782
k_{esc} (min^{-1})	0.668
k_c (min^{-1})	0.199
k_{phos} (min^{-1})	2.590×10^{11}
# Parameters	6
Log likelihood	14.558
BIC	34.534

mRNA Retention Model

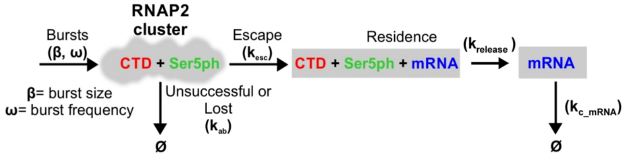

Parameter	Fit
β	14.95
ω (min^{-1})	0.434
k_{ab} (min^{-1})	0.7868
k_{esc} (min^{-1})	0.6728
$k_{release}$ (min^{-1})	0.1987
$k_{c\text{-}mRNA}$ (min^{-1})	6.597
# Parameters	6
Log likelihood	14.561
BIC	34.54

Sup. Fig. 6: **Mathematical models tested.**

Sup. Fig. 6: In the simplest "Two-state Bursting Model", RNAP2 comes in with all 3 signals in a bursting fashion and leaves with a rate k_{c}. "The Two-State Bursting Model k_{off} unfixed" is the same as the previous model, however with the parameter k_{off} not fixed at 1000 and allowed to optimize. The "Chosen Model" is the model described in the main text and in Fig.3. This model was selected as it fits the experimental data well with a minimum amount of parameters (lowest Bayes Information Criterion, BIC, of tested models). The "Fractional Phosphorylation Model" contains an extra parameter compared to the Chosen Model, frac. Here, frac represents the fraction of unescaped RNAP2 with Ser5ph. Frac is analogous to the ratio of two rates with timescales much faster than the rest of the model: CTD gaining Ser5ph and CTD+Ser5ph losing Ser5ph. In the "Phosphorylation Model", RNAP2 binds the promoter and then becomes Ser5ph phosphorylated at a rate of k_{phos}. With this model, RNAP2 requires the Ser5ph signal to escape and transcribe to completion. The "mRNA Retention Model" allows mRNA to remain at the transcription site after RNAP2 completes transcription. All RNAP2 with Ser5ph leave with rate k_{release} and mRNA are retained at the TS. The mRNA then leave at a rate of $k_{\mathrm{c-mRNA}}$. Next to each model, the estimated parameters, maximum likelihood, and BIC are shown.

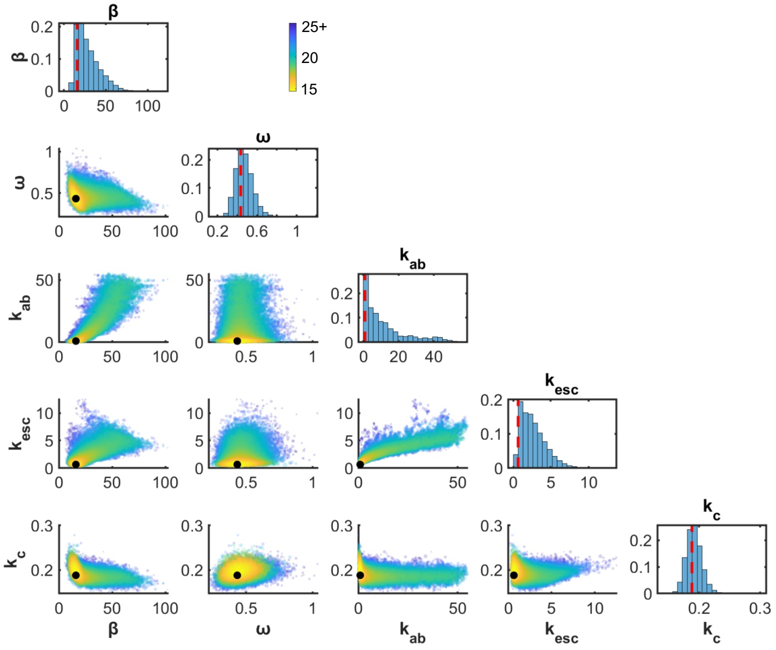

Sup. Fig. 7: **Parameter sensitivity analysis.** Metropolis-Hastings algorithm was run to determine posterior uncertainty of model parameters given the experimental data. Plots on the diagonal show the marginal posterior parameter distributions for each parameter (MLE parameter estimate denoted by red dashed line) and off-diagonal plots show the joint posterior parameter distributions for all pairs of parameters (MLE parameter combination denoted by black marker. Colors denote log-likelihood value; an upper bound of the 0.5% highest log-likelihoods was selected for coloring purposes. Any log-likelihood's color above this threshold was set to that bound). A proposal distribution of a 5 dimensional Gaussian with a standard deviation of 3% of MLE parameters was used. 20 individual chains of 250000 with a thinning rate of 20 (100 million) were used to generate the posterior distributions. 40000 points of the posterior are displayed in the figure.

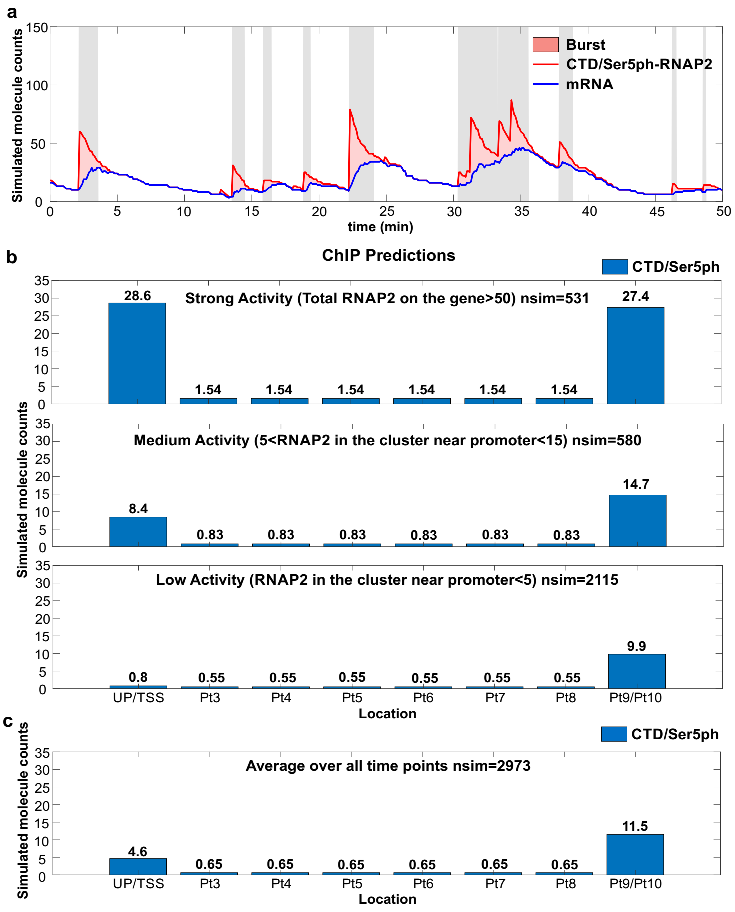

Sup. Fig. 8: **Simulated trajectories and ChIP predictions.** **(a)** Stochastic simulation for the number of nascent mRNA per transcription site (blue line), total number of RNAP2 at transcription site (red line), and number of RNAP2 in cluster near transcription site promoter (red shading). Periods with ≥ 10 RNAP2 at the transcription site cluster (gray shading) are classified as 'ON' (14.0% of total time); periods with no RNAP2 at the cluster are classified as 'OFF' (42.9% of time); and periods with intermediate levels of RNAP2 in the cluster are classified as 'transient' (43.1% of time). Note that for clarity these simulations do no include the experimental shot noise used to simulate actual measurements (as in Fig. 3f, for example). **(b)** Simulated ChIP data as predicted using the model for: (Top; Strong Activity) average spot during an ON period; (Middle; Medium Activity) average spot during a transient period; and (Bottom; Low Activity) average spot during an OFF period. Each stochastic simulation was run for 120,000 min and sampled at 40 min intervals to ensure de-correlated points. To estimate RNAP2 loading at the inner bins, an elongation rate of 4.1 kb/min was assumed and used to get the fraction of time spent elongating versus processing of the total RNAP2 residence time. This fraction of elongation time was then distributed from the final bin uniformly to the middle bins and is represented by the middle numbers of bins Pt3-8. **(c)** Average simulated RNAP2 ChIP over all times including all ON, OFF and transient periods.

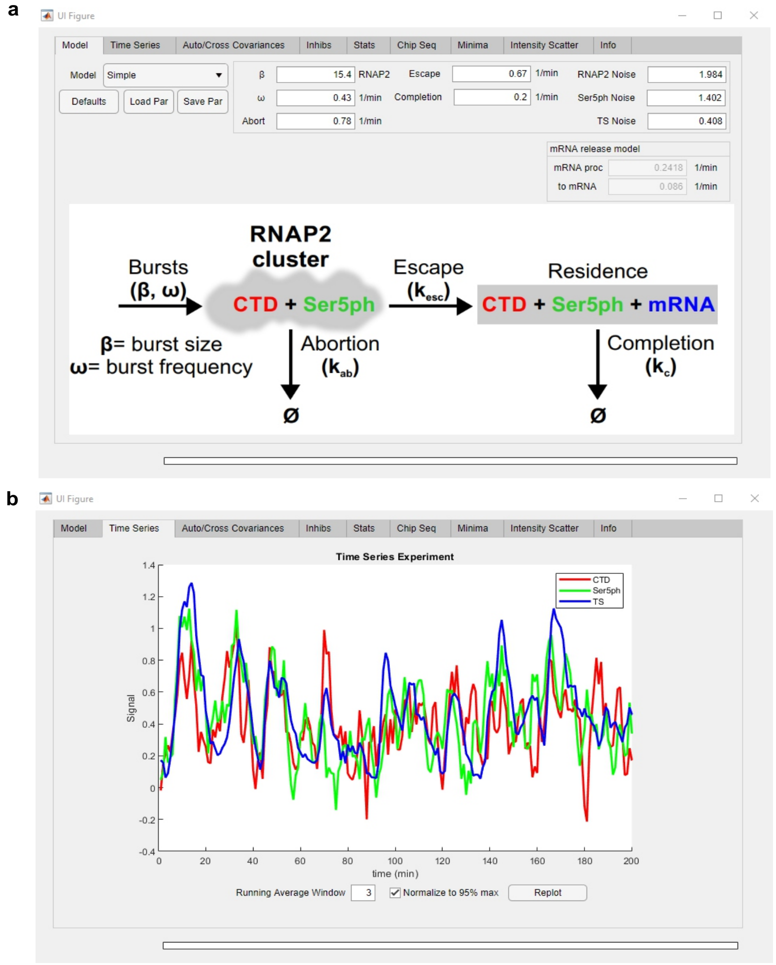

Sup. Fig. 9: **Graphical User Interface (GUI) for the transcription model.** To facilitate the simulation of transcription dynamics at a single-copy gene, the model described in the main text has been incorporated into a MATLAB toolbox. **(a)** This graphical user interface (GUI) is divided into eight upper tabs, and input boxes for specification kinetic parameters. The GUI allows the simulation of intensity trajectories in each channel. **(b)** Sample display of simulated intensities normalized to the 95th percentile and running averaged with a window of three time points. The GUI also allows for display of auto-, cross-correlations, predicted minima from the experimental data previously loaded, prediction of ChIP distributions, and perturbed intensity trajectories by blocking different steps of transcription in the model.

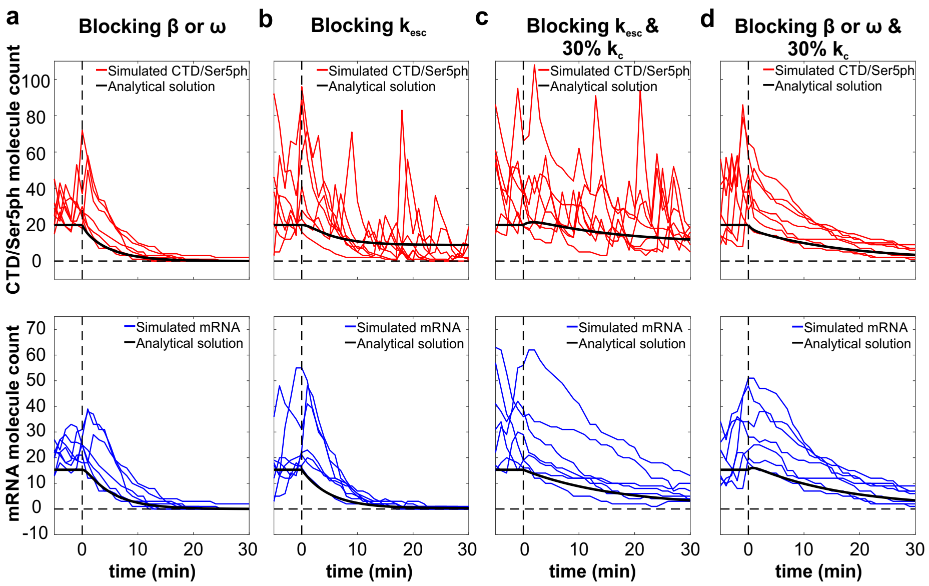

Sup. Fig. 10: **Predicted CTD/Ser5ph-RNAP2, and mRNA signals after perturbing different steps in the mathematical model.** Simulated molecule counts for CTD/Ser5ph-RNAP2 (red, upper panels), and mRNA (blue, bottom panels) after blocking: **(a)** β or ω, **(b)** k_{esc}, **(c)** k_{esc} and 30% k_{c}, and **(d)** β or ω and 30% k_{c}, and their respective analytical solution in each plot (black curve). Simulated trajectories with mRNA molecule counts above the analytical solution at time of inhibition are shown with colored lines. This was done to simulate the experimental procedure of choosing transcription sites at the beginning of an experiment where all three signals could be seen. Blocking is defined as multiplying the best fit parameter by 0.01 (99% reduction), similarly blocking 30% refers to multiplying the best fit parameter by 0.3 (70% reduction). For blocking β and ω, k_{off} was defined by setting k_{off} to 1000, effectively turning off bursting dynamics.)

Table S1: List of primers for ChIP-qPCR as shown in Figure 1. All primer sequences are 5' to 3'.

Name	Forward Primer	Reverse Primer
Up	GGATGACCCGGAGAGAGAAGTG	AAGCAGCTGCTTATATGCAGG
TSS	GCATATAAGCAGCTGCTTTTTGCC	TCCCTGTTCGGGCGCCACT
Pt3	AAGGGAAACCAGAGGAGCTCTC	CCCATCTCTCTCCTTCTAGCCTC
Pt4	CCATCCCTTCAGACAGGATCAGAAG	GCTCTTCCTCTATCTTGTCTAAAGCTTCC
Pt5	GGAGGAGATATGAGGGACAATTGGA	AAGGAACAAAGCTCCTATTCCCACT
Pt6	TGTCTGGTATAGTGCAGCAGCAG	GCTGTTGATCCTTTAGGTATCTTTCCAC
Pt7	CTGTGCCTTGGAATGCTAGTTGGA	TTCTTGCTGGTTTTGCGATTCTTCA
Pt8	TGGGCAAGTTTGTGGAATTGGT	ATGGTGAATATCCCTGCCTAACTCT
Pt9	CAGGCCCGAAGGAATAGAAGAAGA	ATTGCTACTTGTGATTGCTCCATGT
Pt10	GACCAATGACTTACAAGGCAGCT	CCCTGGTGTGTAGTTCTGCCA
