# Supplementary material for: Live-cell imaging reveals the spatiotemporal organization of endogenous RNA polymerase II phosphorylation at a single gene
Source: Nat Commun. 2021 May 26;12:3158. doi: 10.1038/s41467-021-23417-0 (PMC8155019; doi:10.1038/s41467-021-23417-0)
Supplement: Supplementary file 4 — Description of additional supplementary files [file 41467_2021_23417_MOESM4_ESM.docx]

Descriptions of additional supplementary information

Title: Supplementary Movie 1.

Description: Dynamics of the cell presented in Fig. 2a, exhibiting multiple cycles of transcription. Maximum projection of a 13 zstack three-color movie showing an exemplary H-128 cell, in which mRNA (blue), and the RNAP2 Fabs targeting the CTD-RNAP2 (red) and Ser5phRNAP2 (green) at the transcription site (within dashed-white circle) of the HIV-1 reporter gene are co-localized. Raw and bandpass filtered crops for each signal and merge (white) over time are shown in the insets. Images were acquired every 1 min for a total of 200 min, shown here as a moving average (over three time points). At select time points, various signals can be seen in the absence of the others, confirming no fluorescence bleed through, and demonstrating signals are not perfectly synchronous. For example, t=30-40 min shows a strong burst of all three signals, while t=162- 165 min shows CTD-RNAP2 without Ser5ph-RNAP2, t=51-54 min shows Ser5ph-RNAP2 without CTD-RNAP2, and t=149-154 min shows mRNA without the other RNAP2 signals. Scale bar, 10 µm.

Title: Supplementary Movie 2.

Description: Dynamics of the cell presented in Fig. 4a, before and after the addition of 5 µM of TPL. Maximum projection of a 13 z-stack three-color movie showing an exemplary H-128 cell co-localizing mRNA (blue), and the RNAP2 Fabs targeting the CTD-RNAP2 (red) and Ser5phRNAP2 (green) at the transcription site of the HIV-1 reporter gene before TPL application. Images were acquired every 1 min for a total of 35 min, shown here as a moving average (over three time points). All signals quickly disappeared within 10 min of exposure to TPL. Scale bar, 10 µm
