# Supplementary material for: Live-cell imaging reveals the spatiotemporal organization of endogenous RNA polymerase II phosphorylation at a single gene
Source: Nat Commun. 2021 May 26;12:3158. doi: 10.1038/s41467-021-23417-0 (PMC8155019; doi:10.1038/s41467-021-23417-0)
Supplement: Supplementary file 2 — Peer Review File [file 41467_2021_23417_MOESM2_ESM.pdf]

Editorial Note: This manuscript has been previously reviewed at another journal that is not operating a transparent peer review scheme. This document only contains reviewer comments and rebuttal letters for versions considered at Nature Communications.

REVIEWERS' COMMENTS

Reviewer #1 (Remarks to the Author):

The authors have responded to all of my questions and I believe the manuscript is now ready for publication.

Reviewer #2 (Remarks to the Author):

All concerns have been addressed
